# A Patient of Lupus Presenting with Myocarditis and Overlapping Autoimmune Hepatitis

**DOI:** 10.1155/2011/402483

**Published:** 2011-12-12

**Authors:** P. Chattopadhyay, D. Dhua, C. A. Philips, J. Ghosh

**Affiliations:** Department of Medicine, Nil Ratan Sircar Medical College, 138 AJC Bose Road, West Bengal, Kolkata 700014, India

## Abstract

Systemic lupus erythematosus has myriad presentations. Symptomatic myocarditis and/or symptomatic autoimmune hepatitis associated with lupus are rare at presentation. Here we report a young Asian girl, who presented to us with features of symptomatic myocarditis and overlapping autoimmune hepatitis. She was eventually diagnosed to be harboring systemic lupus erythematosus, in whom rigorous management led to gratuitous recovery.

## 1. Introduction

Systemic lupus erythematosus (SLE) can involve the heart in innumerable ways, namely, pericarditis, myocarditis, cardiomyopathy, and heart failure monopolizing almost every anatomic component of the heart. Clinically manifested myocarditis is rare in SLE and is excluded from the diagnostic criteria and disease activity index tool. Symptomatic hepatic involvement in SLE is well documented but considered a rarity. Patients of connective tissue disorders are susceptible for developing autoimmune hepatitis (AIH) and vice versa. Symptomatic myocarditis and autoimmune hepatitis in patients of SLE occurring simultaneously at presentation have not been reported before.

## 2. Case Report

A 20-year-old unmarried female student from rural West Bengal presented to our emergency room with a history of low-grade fever for duration of one month which was not associated with any chills and/or rigors. On further inquiry, she admitted to have symmetrical inflammatory polyarthritis involving small joints of the hands and feet. She also complained of gradually progressive yellowish discoloration of the eyes and mucous membranes since last two weeks. She had sought medical help for her fever two weeks back at a local medical centre and was prescribed oral azithromycin (500 mg) for seven days. Ten days afterwards, her symptoms began to surge, with deepening of jaundice and lethargy and, thence, admitted to our department. During this period, she denied having symptoms of anorexia, nausea, vomiting, weight loss, muscle aches, abdominal pain, pruritus, clay-colored stools, skin rashes, cough, or any bleeding diathesis. The past history was pristine, without any jaundice, blood transfusions and operative interventions, or high-risk behavior. The family history was noncontributory. 

**Figure 1:**
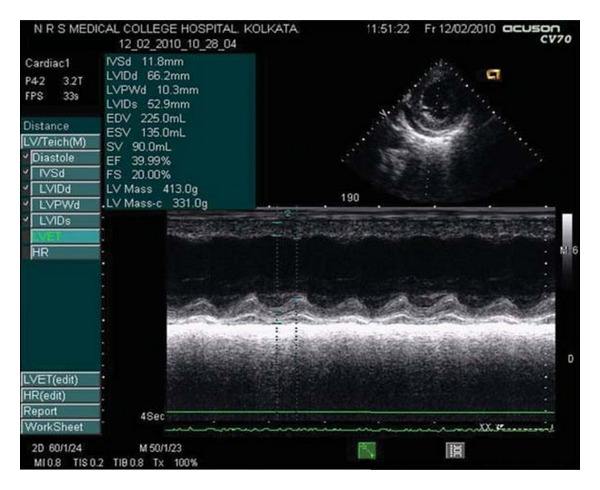
Pretreatment echocardiography showing severely depressed left ventricular systolic function.

General survey revealed an alert patient with pallor and scleral icterus. She had severe nonscarring alopecia without any visible skin rash. She was febrile, with a temperature of 99.5°F. Pulse was feeble but regular at a rate of 120 per minute and was equally palpable in all extremities. Blood pressure was 100/70 mm of Hg in the right brachial region in supine position. There were no associated lymphadenopathy or bone tenderness. The jugular venous pressure was elevated with normal waveforms. Systemic examination revealed nondeforming symmetrical polyarthritis involving small joints of the hands and feet, a soft tender hepatomegaly with a liver span of 15 centimeters, without other organomegaly or ascites. Cardiac examination revealed an ill-sustained left ventricular type of apex at the 6th left intercostal space and a prominent left ventricular third heart sound. Lung auscultation revealed diminished bibasal vesicular breath sounds. The nervous system and musculoskeletal examination was essentially normal. Routine investigations revealed a hemoglobin level of 6.2 gm/dL, normochromic normocytic red blood cells (RBCs), and a total RBC count of 2.6 million/*μ*L. The hematocrit was 38%, and a direct Coomb's test (DCT) came out to be positive. The total leucocyte count was 8400/cmm with a neutrophilic predominance. The thrombocyte count was 70,000/cmm, and the erythrocyte sedimentation rate (ESR) was 120 mm in the 1st hour (the Westergren method). The corrected reticulocyte count was 3.5%. Function tests of the liver (LFT) revealed a total bilirubin of 8.4 mg/dL (with conjugated bilirubin fraction of 4.1 mg/dL), alkaline phosphatase 219 IU/dL, alanine transaminase 274 IU/dL, aspartate transaminase 452 IU/dL, serum albumin 2.4 gm/dL, serum globulin 5.0 gm/dL, and serum lactate dehydrogenase (LDH) 903 IU/L with a fasting blood glucose of 80 mg/dL, serum blood urea nitrogen of 38 mg/dL, serum creatinine of 0.8 mg/dL, serum uric acid 6.3 mg/dL, serum sodium and potassium 141 meq/L and 5.1 meq/L, respectively. A fasting lipid profile was within normal limits. Serum creatine phosphokinase (CPK-MB fraction) and troponin T were normal. The urinalysis revealed a sterile urine with a pH of 7.1, specific gravity 1.010, which was positive for albumin and having 7–10 pus cells/high-power field with occasional granular casts. The 24-hour urinary protein excretion was 650 mg/dL. An electrocardiogram revealed sinus tachycardia with inverted T waves in all the precordial leads. The chest skiagram taken in postero-anterior view showed presence of bilateral pleural effusion. Abdominal ultrasound revealed an echogenic liver with span of 16 cm without splenomegaly or abdominal adenopathy but confirmed the presence of bilateral pleural effusion which was more on the right side and the presence of small amount of ascites. A pleurocentesis from the right hemithorax and subsequent fluid analysis revealed a transudative effusion. The viral markers for hepatitis were negative. A workup on Wilson's disease came negative. The prothrombin time was 12.7 seconds (control 11.1 seconds). Initial supportive measures were under taken to relieve the patient of her symptoms. On the 3rd and 4th days of hospital stay, she suffered two episodes of acute shortness of breath. Her blood pressure at that time was 90/62 mmHg in the right brachial region and was followed by appearance of pink frothy sputum, which responded promptly to intravenous furosemide and inhaled moist oxygen. The respiratory distress was relieved to some extent, but the patient continued to have shortness of breath on mild physical activity (New York Heart Association Grade 3) along with palpitations. The clinical examination revealed a persistence of gallop rhythm. A transthoracic echocardiography (TTE) revealed global left ventricular hypokinesia and dilated left ventricle with poor left ventricular function with an ejection fraction of 39.99% and fractional shortening of 20%. A Grade 1 mitral regurgitation was detected with mild pericardial effusion, without signs of tamponade. There were no features suggestive of pulmonary arterial hypertension, vegetations, or clots (Figure 1).

**Figure 2:**
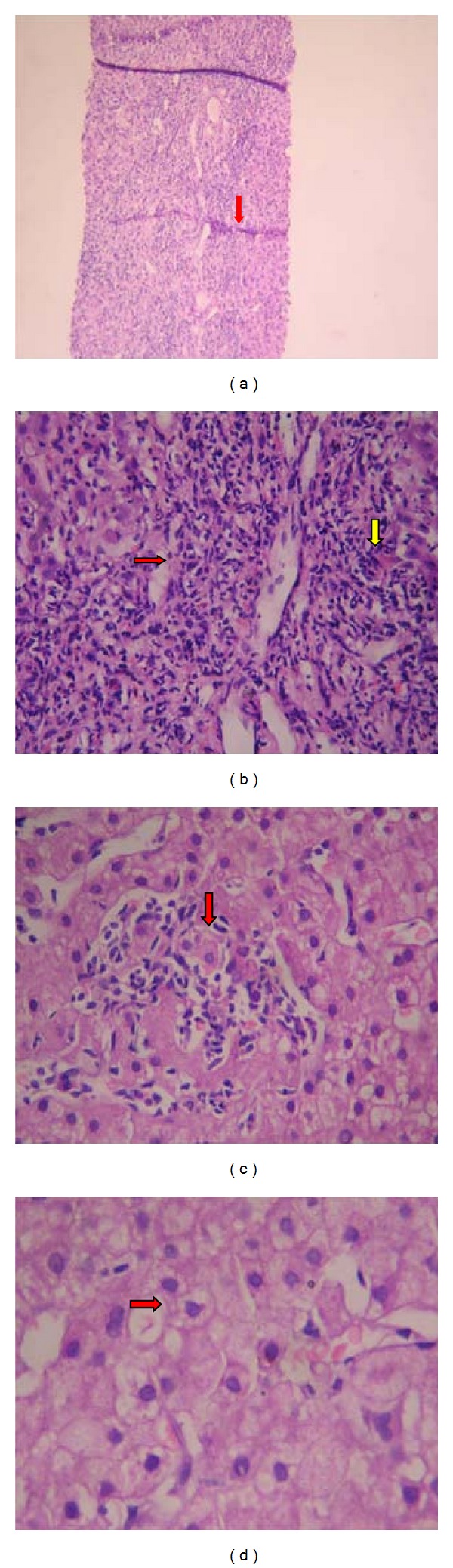
(a) Mild distortion of architecture by enlarged portal areas and bridging fibrosis/necrosis (red arrow) [Hematoxylin and Eosin Stain, 50x]. (b) Interface hepatitis (red arrow) and portal inflammation (yellow arrow) [Hematoxylin and Eosin Stain, 100x]. (c) Lobular inflammation (red arrow) [Hematoxylin and Eosin Stain, 150x]. (d) Hydropic hepatocytes with attempted rosette formation (red arrow) [Hematoxylin and Eosin Stain, 200x].

The engrossing way these series of events unfolded prompted us in undertaking further investigations, which revealed an antinuclear antibody (ANA) to be positive at 1 : 320 titre with homogeneous immunofluorescence pattern. The subsequent antibody to double-stranded DNA was positive at 1 : 20 titer (Crithidia Method), and anti-Ro antibody was positive at 135.65 U/mL. The anti-Smith antibody was negative. The antibody to smooth muscle was positive. The antiphospholipid antibody (APLA) workup came out to be negative. The serum complement levels were low, with C3 levels at 37 mg/dL and C4 at 46 mg/dL. A liver biopsy was done at this point under high-risk precautions to substantiate the underlying hepatopathy. The revelation was that of interface hepatitis, lobular and portal inflammation with plasma cells, and hydropic changes in some of the hepatocytes with attempted rosette formation; changes that were characteristic of autoimmune hepatitis (AIH) (Figures 2(a), 2(b), 2(c), and 2(d)).

**Figure 3:**
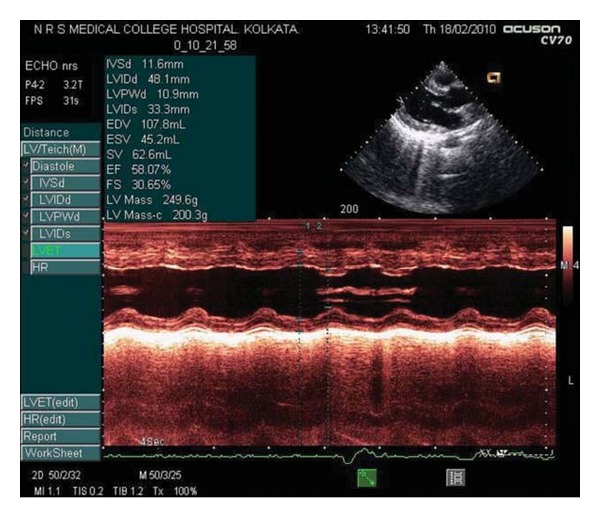
Posttreatment echocardiography showing marked improvement of left ventricular systolic function.

A diagnosis of Lupus myocarditis with autoimmune hepatitis was made, and the patient was started on intravenous methyl prednisolone at a dose of 1 gm/day for 5 consecutive days, followed by oral prednisolone at a dose of 1 mg/kg body weight. Loop diuretics, low-dose angiotensin converting enzyme inhibitor with salt and fluid restriction, was continued throughout. Features of breathlessness, palpitations, and tachycardia improved from the third day itself. A repeat echocardiography done after six days showed marked improvement of the TTE parameters—global left ventricular (LV) function improved with ejection fraction of 58.07% and fractional shortening of 33.65%. In comparison to the previous echocardiographic profile, there were no mitral regurgitation or pericardial effusion in the posttreatment period (Figure 3). The constitutional symptoms subsided, and the hematological parameters started to improve in two weeks, with the liver function tests normalizing in due course, over the following five weeks.

## 3. Discussion

Our patient satisfied the American College of Rheumatology criteria for diagnosis of SLE as well as criteria for autoimmune hepatitis. Although earlier necropsy studies have estimated the prevalence of myocarditis in SLE to be 50–80%, most of those cases were subclinical. Symptomatic SLE myocarditis is rare and occurs in up to 9% of cases [1, 2]. Endomyocardial biopsy remains the gold standard for diagnosis, but it has its pitfalls—an invasive process with associated complications—and there is a low sensitivity and specificity associated with it (due to the patchy nature of myocardial involvement in lupus myocarditis) [3–5]. Thus, for urgent treatment-related decision making, clinical diagnosis of SLE myocarditis remains an important tool as untreated cases may develop abrupt and life-threatening complications including arrhythmias, dilated cardiomyopathy, and heart failure [4, 6]. Heart failure is the most common presenting feature of myocarditis [6]. Pulmonary infection, ischemic heart disease, pulmonary embolism, alveolitis, pulmonary hemorrhage, and pulmonary hypertension can mimic the symptoms of lupus myocarditis-related heart failure like shortness of breath, chest pain, palpitations, pedal edema, and exertion intolerance. Echocardiography, a noninvasive and highly useful adjunct in the proper clinical setting, has many parameters that reportedly have a high index of sensitivity and specificity for diagnosis of acute myocarditis. These include decreased ejection fraction, increased chamber size, decreased diastolic descent rate of anterior mitral leaflet, decreased ratio of mean systolic to mean diastolic velocity of left ventricular posterior wall, reduced early to late diastolic flow velocity (*E/A*) ratio, lower deceleration rate of early diastolic flow velocity, prolonged isovolumetric relaxation time, and atrial ejection force [2, 5, 7]. Our patient met with many of the above mentioned criteria. Viral and ischemic cardiomyopathy were the two other important differential diagnoses considered and then discarded in our case, although viral serology (except for the hepatitis viruses) and angiography were not done. Our patient was a young girl without having any obvious risk factors for atherosclerosis and with an SLE disease activity index of 24 at presentation. In a resource-poor country like ours, we drew inspiration from the same approach in an earlier landmark Asian case series [6]. Anti-Ro, anti-ds-DNA, and APLA positivity has been variably associated with SLE myocarditis [1, 6, 8]. Anti-Ro and anti-ds-DNA were positive, and APLA workup negative in our patient. Acute mode of onset, presence of renal and hematological involvement with low complement (C3 and C4) levels, hypoalbuminemia, and raised ESR associated with clinical myocarditis, as found in our case, has been documented rarely [6, 9]. The first presentation of SLE in the form of heart failure, overlapping with autoimmune hepatitis and immune hemolytic anemia as found in our case, is rarer [10, 11]. Anti-Ro is very rarely positive in normal individuals or hospitalized patients with nonrheumatic disorders [12]. Anti-Ro antibody may be associated with cardiac disorders in adults, as well as in neonates [13]. Troponin T, a serum marker with high sensitivity and specificity for cardiac myocyte injury, was negative in our case, as was CPK-MB fraction and troponin T. Troponin T positivity occurs in only 34% of cases and that too, in early cases of autoimmune myocarditis, presenting within a month. CPK-MB is inferior as it is positive in only 5.7% of biopsy-proven cases [14]. This young girl presented to us after her symptoms were present for more than a month, possibly explaining the negativity of CPK-MB and troponin T. Blockade of small vessels of the heart as part of widespread microvascular occlusion in the setting of APLA positivity is another possibility of cardiac dysfunction in SLE. Widespread microvascular thrombosis of cardiac vessels and other organs, culminating into diffuse cardiomyopathy, heart failure, or even cardiac arrest, can occur acute or as chronic manifestation without signs of inflammation or large vessel involvement in patients of SLE. Diagnosis is possible only with an endomyocardial biopsy [15]. However, in the light of APLA and lupus anticoagulant negativity and prompt response to immunosuppression, this diagnosis was less likely in our case on clinical grounds, even though biopsy was not performed. The jaundice and hepatic derangements in a case like ours may be due to SLE activity itself, due to autoimmune hepatitis with other nonhepatic autoimmune manifestations or SLE with coexistent autoimmune hepatitis. Patients with AIH are more prone to develop systemic autoimmune diseases and viceversa [16]. Both SLE and AIH have many autoimmune features in common, namely, polyarthralgia, hypergammaglobulinemia, and ANA positivity [17]. It is difficult, but important the to distinguish between SLE with hepatic dysfunction and AIH from, therapeutic point of view as SLE will lead to renal end organ damage while AIH patients will have hepatic failure as the terminal event. There are some clinical, serological, and, most importantly, histological pointers, which differentiates the two conditions. Previously thought to be rare, hepatic involvement in SLE is now considered to be more clinically significant [18]. Hepatomegaly is common, and elevated liver enzymes can be found in 23.5% cases [19, 20]. The most common histological findings in SLE are fatty infiltration followed by atrophy and/or necrosis of central hepatic cells. In most cases, this concomitant hepatic dysfunction is subclinical [20]. The histological hallmark of AIH is interface hepatitis and portal inflammation with plasma cell infiltration [21]. Concomitant periportal piecemeal necrosis, variable lobular hepatitis, and rosette formation of the hepatic cells further supports the diagnosis of AIH but does not exclude SLE. Presence of only lobular hepatitis tilts the diagnostic scale more towards SLE [17, 22, 23]. Anti-ribosomal-P antibody positivity occurs in 44% of patients with SLE-associated hepatic dysfunction but is absent in AIH, making it a useful serological differentiator between the two [24]. Anti-ds-DNA may be transiently elevated in AIH, though more recent data emphasizes its presence to be associated with active lupus rather than AIH, where it is negative [25, 26]. Anti-Smith antibody may also be positive in SLE-AIH overlap in some cases [27]. In our patient, presence of low serum complement (C3 and C4), DCT-positive hemolytic anemia, ANA positivity, and high titres of ds-DNA and anti-Ro fulfilled the revised ACR diagnostic criteria and pointed strongly towards SLE. Likewise, our patient had jaundice with high AST/ALT ratio, hypergammaglobulinemia, ANA, and anti-smooth muscle antibody positivity with supportive evidence of liver biopsy showing interface hepatitis, periportal piecemeal necrosis, rosette formation, and lobular hepatitis, strongly suggesting the diagnosis of autoimmune hepatitis [28]. Portal plasma cell and lymphocyte infiltration is said to be characteristic of untreated AIH as both lupus hepatitis and treated AIH may have lymphocytic infiltration of the portal tracts in common. A good response to corticosteroids further bolstered the diagnosis of autoimmune hepatitis in our case. AIH-SLE overlap has rarely been reported before [24, 27, 29–32]. This disease entity responds rapidly to steroid therapy with the hepatic dysfunction improving in parallel with stabilization of other systemic manifestations, and the prognosis is generally good [27, 30]. Some researchers have reported less favorable steroid response in AIH-SLE overlap syndrome [32]. We believe that our patient had an overlap of SLE and AIH which is very rare at the outset. A presentation of lupus myocarditis with AIH has not been reported before to the best of our knowledge. Lupus myocarditis must be considered in suspected lupus patients with unexplained tachycardia and acute episodes of breathlessness, as timely intervention with systemic steroid therapy is often life salvaging.
